# A novel collagen area fraction index to quantitatively assess bowel fibrosis in patients with Crohn’s disease

**DOI:** 10.1186/s12876-019-1100-3

**Published:** 2019-11-11

**Authors:** Xue-hua Li, Zhuang-nian Fang, Tian-ming Guan, Jin-jiang Lin, Can-hui Sun, Si-yun Huang, Ren Mao, Bao-lan Lu, Qing-hua Cao, Shi-ting Feng, Zi-ping Li

**Affiliations:** 1 Department of Radiology, The First Affiliated Hospital, Sun Yat-Sen University, 58 Zhongshan II Road, Guangzhou, 510080 People’s Republic of China; 2 Department of Radiology, Hui Ya Hospital of Hui Zhou, Sun Yat-Sen University, 186 Zhongxingbei Road, Huizhou, 516081 People’s Republic of China; 3 Department of Gastroenterology, The First Affiliated Hospital, Sun Yat-Sen University, 58 Zhongshan II Road, Guangzhou, 510080 People’s Republic of China

**Keywords:** Crohn’s disease, Fibrosis, Collagen fiber

## Abstract

**Background:**

A validated histopathological tool to precisely evaluate bowel fibrosis in patients with Crohn’s disease is lacking. We attempted to establish a new index to quantify the severity of bowel fibrosis in patients with Crohn’s disease-associated fibrostenosis.

**Methods:**

We analyzed the histopathological data of 31 patients with Crohn’s disease strictures undergoing surgical resection. The most representative sections of resected strictured segments were stained with Masson trichrome to manifest bowel fibrosis. The collagen area fraction and histological fibrosis score were simultaneously calculated for the same section to evaluate the severity of bowel fibrosis.

**Results:**

Collagen area fraction strongly correlated with histological fibrosis scores (*r* = 0.733, *P* < 0.001). It showed a stronger correlation **(***r* = 0.561, *P* < 0.001**)** with the degree of bowel strictures than the histological fibrosis score did **(***r* = 0.468, *P* < 0.001). It was also shown to be more accurate for diagnosing Crohn’s disease strictures (area under the receiver operating characteristic curve = 0.815, *P* < 0.001) compared with the histological fibrosis score (area under the curve = 0.771, *P* < 0.001). High repeatability was observed for the collagen area fraction, with an intraclass correlation coefficient of 0.915 (*P* < 0.001).

**Conclusions:**

Collagen area fraction is a simple and reliable index to quantify the severity of bowel fibrosis in patients with Crohn’s disease-associated fibrostenosis.

## Background

Approximately 60% of patients with Crohn’s disease (CD) will undergo surgery during their lifetime due to poor response to therapy or the development of strictures [1, 2]. CD stricture is characterized by bowel wall thickening and luminal narrowing that results in bowel obstruction, which is a complication of chronic and recurrent intestinal inflammation and a hallmark of severe CD. Clinically, inflammation-predominant strictures can be relieved by anti-inflammatory medical treatment, whereas fibrosis-predominant strictures require surgical treatment [3]. Because an accurate assessment of bowel fibrosis to distinguish the type of CD stricture has therapeutic implications, many technologies have been applied to address this problem. However, there are currently no established biomarkers including gene variants, serum microRNAs, serum growth factors, serum anti-microbial antibodies, serum extracellular matrix proteins, enzymes, or circulating cells, that have been proven to be strictly specific for fibrostenosis [4]. Among many detection methods, the evaluation of transmural collagen fiber deposition using histopathology, which is commonly used as a reference standard in various studies on CD, is the most reliable and direct method to assess bowel fibrosis.

However, the systematic, histopathological characterization of fibrostenosis in CD patients has been limited [5]. Most of the histopathological assessments that focused on this issue were performed as a part of radiological studies [6–11] with various histological grading systems that evaluated bowel fibrosis. In these studies, bowel fibrosis was roughly and semi-quantitatively scored according to the deposition depth of collagen fiber within each layer on a microscopic examination, with or without intestinal stricture and/or a proximal dilated lumen on macroscopic evaluation [6–11]. There is little doubt that this type of histopathological grading is feasible due to its emphasis on the deposition depth of collagen fiber in the bowel wall. However, the severity of fibrostenosis in CD patients that is mainly induced by excessive collagen fiber deposition is influenced not only by the deposition depth but also by the amount of collagen fiber. On the other hand, to verify the utility of cross-sectional imaging to detect the changes on intestinal fibrosis and the potential efficacy of anti-fibrotic drugs, it is more crucial to quantify the amount of fiber in bowel lesions using histopathology.

However, a detailed histopathological analysis method that focuses on quantifying the amount of collagen fiber in the bowel wall has not been fully and systematically characterized. Hence, the purpose of this study was to develop and verify a simple and reliable histopathological index to quantify bowel fibrosis in patients with CD using a novel imaging segmentation technology. We compared this index with a published semi-quantitatively histopathological grading scheme [10].

## Methods

### Patients

This prospective study was approved by the ethical review board of our institution, and written informed consent was obtained from all CD patients who were treated with surgical resection.

From July 2014 to April 2017, 31 consecutive adult patients with an established diagnosis of CD who were scheduled for elective surgery were enrolled in this study. All patients had long-standing CD that was complicated by bowel obstruction secondary to well-identified strictures, and they received various standard medical treatments before surgery. The exclusion criteria included patients with tissue samples that did not contain all layers of the bowel wall.

### Macroscopic Evaluation.

For each patient, one or more tissue samples were taken from the strictured region or the predominantly thickened bowel wall in fresh resected bowel segments, according to the number and extent of the lesions. The macroscopic findings of all of the obtained tissue samples included the site of the pathological bowel segment and the degree of bowel stricture. Strictures were defined as bowel narrowing that decreased the luminal diameter by at least 50% compared with a normally distended proximal segment [6]. The degree of bowel narrowing or stricture was semi-quantitatively scored as 1 (luminal narrowing < 50% but the criteria for stricture were not met), 2 (stricture without a dilated proximal bowel), or 3 (stricture with a dilated proximal bowel).

### Microscopic Assessment

**Fig. 1:**
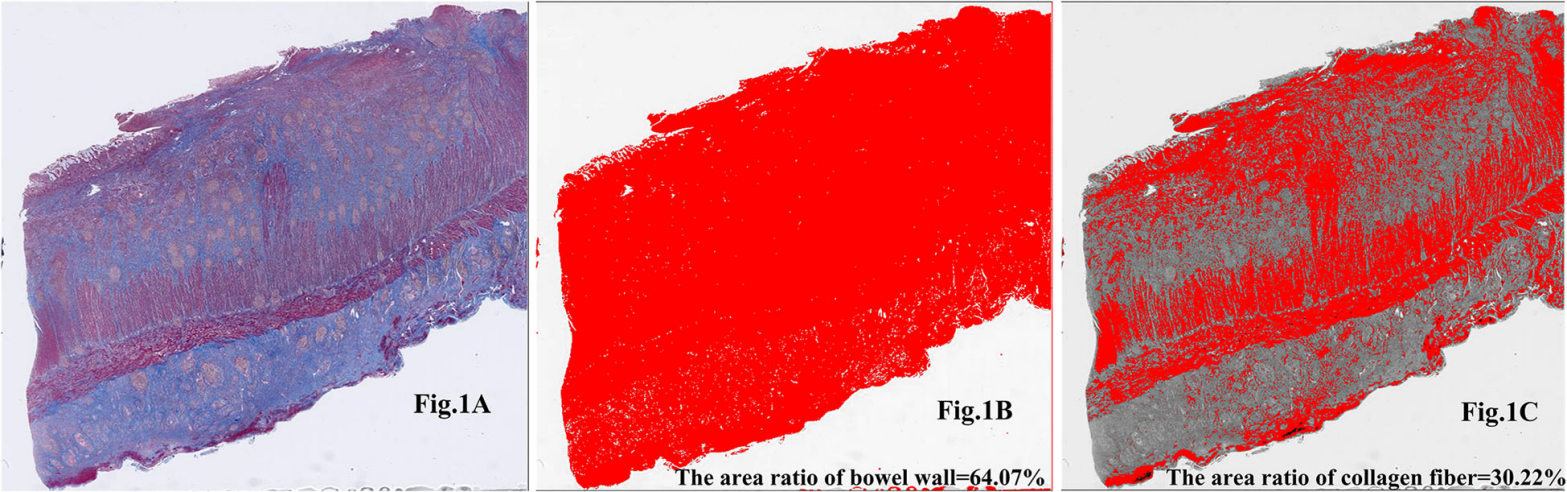
The collagen area fraction for severe fibrostenosis was calculated as a histological fibrosis score of 4 in patients with colonic CD. **a** The fibrotic areas in the bowel wall that were stained using Masson trichrome are shown as a distinct blue region, and the blood, muscle, and inflammatory cells are shown in red. **b** The area of the bowel wall and (**c**) the area of collagen fiber within the bowel wall are automatically identified and divided by the area of the whole picture to the yield area ratio. The collagen area fraction = [30.22% / 64.07%] × 100% = 47.17%. CD, Crohn’s disease

After the tissue was fixed in formalin, a full-thickness, well-oriented, and transverse sample of the resected bowel segment was embedded in paraffin and sliced into a 4-μm-thick section. The section was stained with Masson’s trichrome to assess histological fibrosis using a semi-quantitative and quantitative analysis method. The fibrotic areas that were stained with Masson trichrome were shown as a distinct blue region, and the blood, muscle, and inflammatory cells appeared in red (Fig. 1). A pathologist (Q.C., who had 9 years of experience in digestive tract pathology and did not know the patient’s clinical information) microscopically scored the severity of bowel fibrosis observed on histological sections from the most severely affected areas. The pathologist scored the fibrosis from 0 to 4 using a semi-quantitative scoring system that was previously described (Table 1) [10]. The time to score the bowel fibrosis for a section was approximately 1 min.

**Table 1:** Histologic score for fibrotic CD

Score	Fibrosis
0	No fibrosis
1	Minimal fibrosis in submucosa or subserosa
2	Increased submucosal fibrosis, septa into muscularis propria
3	Septa through muscularis propria, increase in subserosal collagen
4	Significant transmural scar, marked subserosal collagen

*CD* Crohn’s disease

### Imaging Manipulation and Quantitative Analysis of Bowel Fibrosis

Digital images were created from all sections using a whole-slide scanner (KF-PRO-005, Konfoong Biotech International CO., Ltd., Ningbo, China). Two observers (X. L. and Z.F., with 7 and 5 years of experience in CD research, respectively) without knowledge of the patients’ clinical information and histological scoring digitized and quantified the images using public domain software (Image J program, developed at the National Institutes of Health and available at: https://imagej.nih.gov/ij/download.html). This software enables cross-sectional image segmentation and quantitative analysis of the properties of the bowel wall’s tissue.

Using the Image J software, the researchers sketched and marked the whole full-thickness bowel wall, excluding the mesenteric fat first. Then, the area of the bowel wall was divided by the area of the whole picture (including the white background and colorful bowel wall) to automatically yield the area ratio of the bowel wall. Subsequently, collagen fiber in the bowel wall was automatically identified and differentiated from other tissue properties, including blood, muscle, and inflammatory cells, after converting the color images into gray-colored figures (Fig. 1). To sketch the area of collagen fiber more precisely, the researchers sometimes needed to slightly adjust the contrast between the collagen fiber and other tissue components manually. Similarly, the area of the collagen fiber was divided by the area of the whole picture to automatically yield the area ratio of the collagen fiber. Hence, the ratio between the area of the collagen fiber and the total area of the bowel wall was calculated as follows: Collagen area fraction [%] = [Area ratio of collagen fiber / Area ratio of the bowel wall] × 100%. We randomly selected the results of one of the two observers for further analysis. The time to sketch a bowel wall and then calculate a collagen area fraction was 1 to 3 min. Approximately one third of the sections needed to be slightly adjusted for image contrast, with an average time of 30 s.

### Statistical analysis

The statistical analysis was performed using two-sided comparisons, and significance was defined as a *P*-value < 0.05. We used SPSS software, version 20.0 (SPSS Inc., Chicago, IL, USA). Quantitative data are expressed as the mean ± standard deviation, and qualitative data are presented as a percentage and/or absolute value. Bivariate correlations between the different parameters were analyzed using Spearman’s rank correlation. A correlation coefficient (*r*) < 0.01 was considered as none, 0.01–0.24 as minimal, 0.25–0.49 as fair, 0.50–0.74 as moderate to good, and 0.75–1.00 as very good to excellent. A receiver operating characteristic (ROC) curve analysis was performed, and the area under the ROC curve (AUC) was calculated to determine the diagnostic accuracy of the collagen area fraction and histological fibrosis score for CD strictures. The optimal threshold was determined using a ROC curve analysis following Youden’s index. An intra-class correlation coefficient (ICC) was applied to test the inter-observer agreement. An ICC < 0.20 was considered as poor, 0.21–0.41 as fair, 0.41–0.60 as moderate, 0.61–0.80 as good, and 0.81–1.00 as excellent. A Bland-Altman plot was generated to visually demonstrate the agreement of measurement values between the two observers. In the Bland–Altman analysis, 95% limits of agreement (LoA) were defined as the mean difference ± 1.96 × the standard deviation.

## Results

### Demographic and Clinical Data

Of 88 specimens from the 31 patients (19 men, 12 women, mean age, 31.23 ± 7.95 years) who were enrolled in the study, two specimens were excluded from the analysis because the bowel wall was incomplete. The remaining 86 specimens with acceptable pathological staining quality were included in this study. For each patient, histological slices were available from 1 to 3 bowel lesions. Of the 86 specimens, 54 were from the most stenosed areas or prominently thickened bowel walls in the ileum (*n* = 44) and jejunum (*n* = 10), and 32 were from lesions in the colon. The demographic and clinical characteristics of the patients are summarized in Table 2.

**Table 2:** Baseline Demographic and Clinical Characteristics of the Patients

	*N* = 31
Gender: male / female	12 /19
Age, range, years	18–63
Disease duration, mean ± SD, months	66.45 ± 63.77
Smoking history	
Never	21 (67.74%)
Current smoker	4 (12.90%)
Former smoker	6 (19.35%)
Therapy at the time of surgery	
Anti-TNF and immunosuppressant or corticosteroid	11 (35.48%)
Anti-TNF	14 (45.16%)
immunosuppressant	6 (19.35%)
Surgery type, *n* (%)	
Ileocolon resection	19/31 (61.29%)
Partial small bowel resection	8/31 (25.81%)
Partial colon resection	4/31 (12.90%)
Regions of disease involvement	
Ileum only	5/31 (16.13%)
Ileum + jejunum	3/31 (9.68%)
Ileum + jejunum+colon	4/31 (12.90%)
Ileum + colon	15/31 (48.39%)
Colon only	4/31 (12.90%)
CDAI, mean ± SD	232.12 ± 73.65
CRP, mean ± SD, mg/L	42.07 ± 21.19
ESR, mean ± SD, mm/h	41.37 ± 19.34

*TNF* Tumour necrosis factor; *CDAI* Crohn’s disease activity index, *CRP* C-reactive protein, *ESR* Erythrocyte sedimentation rate

### The Correlation Between the Collagen Area Fraction and Histological Fibrosis Score

**Fig.2:**
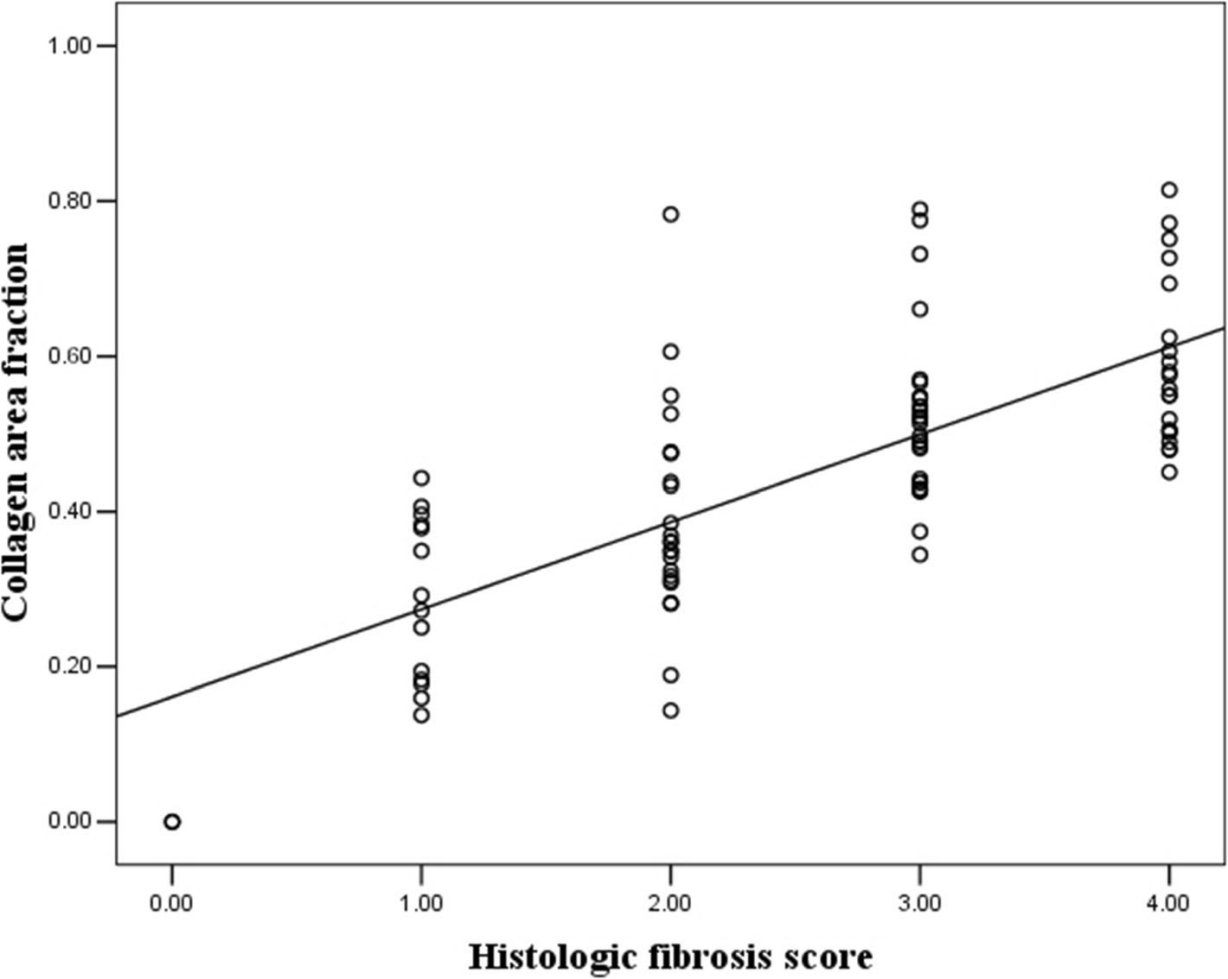
A scatterplot shows that there was strong correlation between the collagen area fraction and histological fibrosis score (*r* = − 0.733, *P* < 0.001)

Histological fibrosis on Masson trichrome staining was scored as 0 (*n* = 2), 1 (*n* = 14), 2 (*n* = 23), 3 (*n* = 26), or 4 (*n* = 21). The mean collagen area fraction was 0.45 ± 0.17, with a range of 0 to 0.81. There was a good correlation between the collagen area fraction and histological fibrosis score (*r* = 0.733, *P* < 0.001) (Fig. 2). In specimens with non-severe fibrosis (score 0–2), 89.74% (35/39) of the specimens were shown to have a collagen area fraction ≤50%, whereas 10.26% (4/39) of the specimens had a collagen area fraction > 50%. In specimens with severe fibrosis (score 3–4), 63.83% (30/47) of the specimens had a collagen area fraction > 50%, while 36.17% (17/47) of them had a collagen area fraction ≤50%.

### The Collagen Area Fraction Versus the Histological Fibrosis Score in Assessing the Degree of CD Strictures

**Fig. 3:**
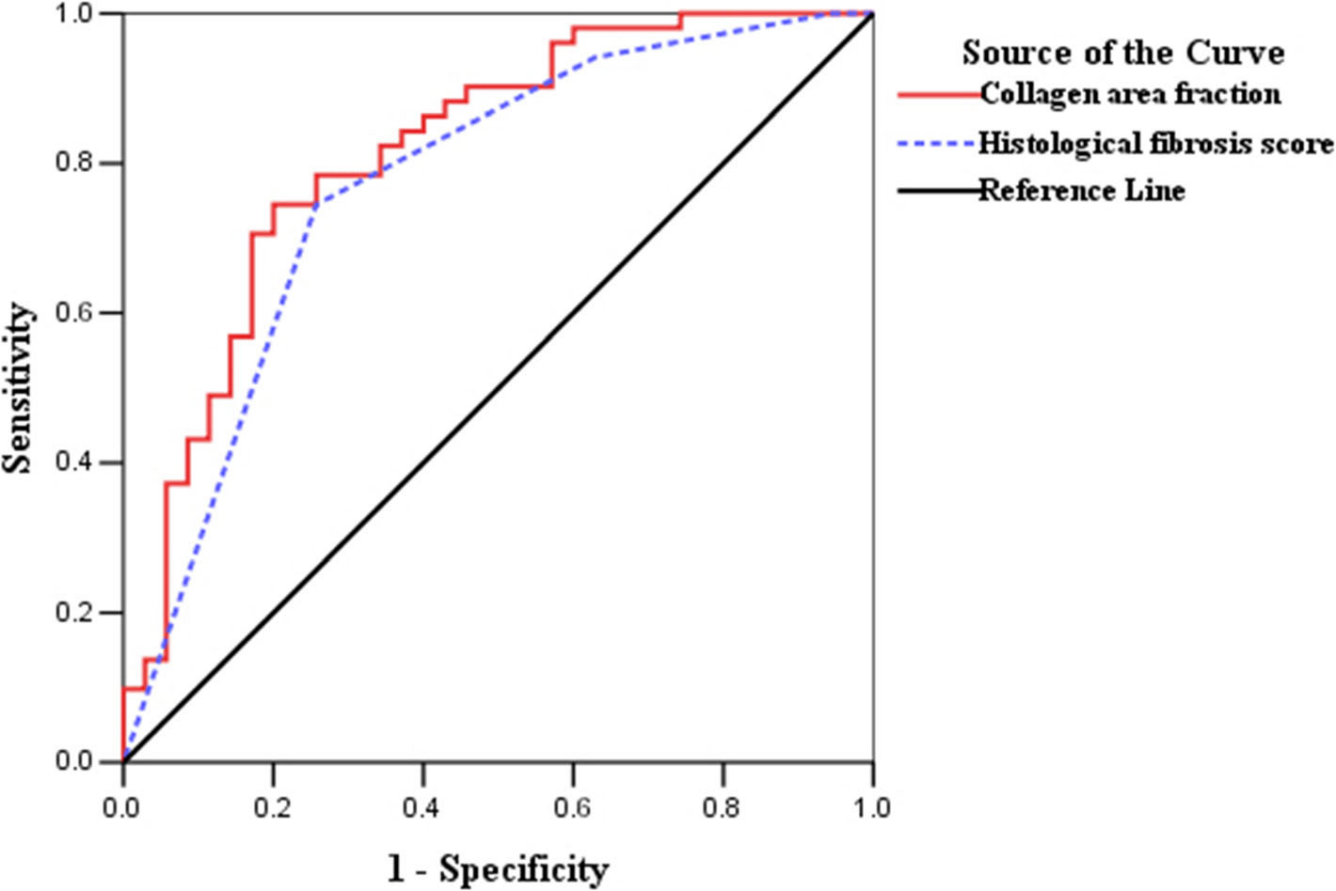
On the ROC analysis, the collagen area fraction was slightly more accurate, with an AUC of 0.815 (95% CI = 0.721–0.910, *P* < 0.001), than the histological fibrosis score (AUC = 0.771; 95% CI = 0.666–0.877; *P* < 0.001) for diagnosing CD strictures. ROC, receiver operating characteristic; AUC, area under the ROC curve; CD, Crohn’s disease; CI, confidence interval

The degree of strictures of the 86 bowel segments that were examined in this study was scored as 1 (*n* = 35), 2 (*n* = 40), or 3 (*n* = 11). The collagen area fraction **(***r* = 0.561, *P* < 0.001**)** showed a stronger correlation with the degree of stricture than the histological fibrosis score **(***r* = 0.468, *P* < 0.001). The ROC curve analysis demonstrated that the collagen area fraction had a slightly higher accuracy (AUC = 0.815; 95% confidence interval [CI] = 0.721–0.910; *P* < 0.001) for diagnosing CD strictures (score 2–3) than the histological fibrosis score (AUC = 0.771; 95% CI = 0.666–0.877; *P* < 0.001) (Fig. 3). Using the collagen area fraction of 0.45 as a cutoff value, we found that the sensitivity and specificity of this index were 74.50 and 80%, respectively.

### Inter-Observer Agreement

**Fig.4:**
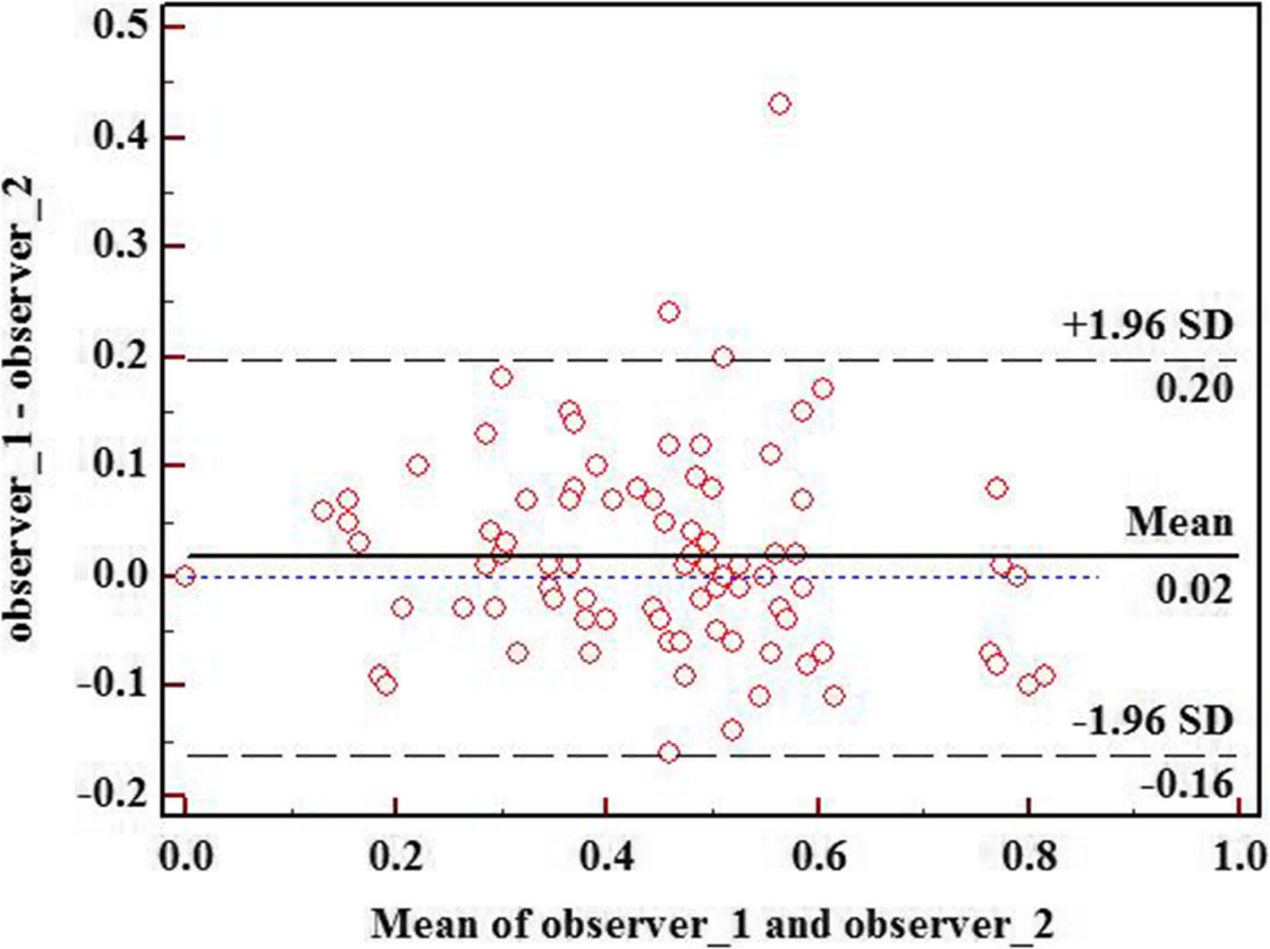
A Bland–Altman analysis of the difference between the two observers for measuring the collagen area fraction. The Bland-Altman plot shows the LoA. The mean difference is shown as a solid line, the upper and lower 95% LoA are shown as black dashed lines, and the zero difference is shown as the blue dashed line. LoA, limits of agreement

The repeatability of the collagen area fraction between the two observers was excellent, with an ICC of 0.915 (*P* < 0.001). In the Bland-Altman plots (Fig. 4), the mean difference of the collagen area fraction between the two observers was 0.02, and the 95% LoA was − 0.16 to 0.20 (95% CI = -0.20 to 0.23).

## Discussion

The most important findings in our study were that the collagen area fraction, which was calculated as the ratio between the area of the collagen fiber and the total area of the bowel wall, showed not only a good correlation with the histological fibrosis score in detecting bowel fibrosis but also a higher accuracy for diagnosing CD strictures than the histological fibrosis score. We also demonstrated that the collagen area fraction, which was measured with Image J software, had high repeatability for quantifying the amount of collagen fiber that was deposited in the bowel wall.

Fibrosis in CD patients can involve the full-thickness bowel wall, including the mucosa, submucosa, muscularis propria, and serosa layers. It results from an abnormal response to chronic injury in the bowel wall and is characterized by excessive deposition of extracellular matrix proteins [12]. Bowel fibrosis is a major contributor to the failure of medical treatment and hospitalization for surgical resection. Intestinal fibrosis has changed from being a static and irreversible entity to a dynamic and reversible disease [13, 14]. Hence, many promising biomarkers or imaging technologies have aimed to detect bowel fibrosis to initiate new antifibrotic agents and predict intestinal fibrosis. To verify the efficacy of these biomarkers or technologies to treat this clinically challenging condition, histological findings are usually used as the most reliable reference standard. The choice of the histological reference standard is particularly important, because an improper reference standard may significantly influence the diagnostic accuracy of the investigated index.

However, no validated histopathological evaluation system is available to grade the severity of bowel fibrosis [14]. Most of the histopathological assessments that focused on this issue were performed as a part of radiological studies [6–11]. Adler et al. [9, 10] used a histopathological grading system to assess bowel fibrosis in radiological studies, in which the severity of bowel fibrosis was semi-quantitatively scored based on the depth of deposition of collagen fibers from the submucosa to subserosa. Another histopathological grading system that was widely used in CD studies semi-quantitatively scored the severity of bowel fibrosis based on the deposition depth of collagen fiber, muscular hyperplasia, and the degree of intestinal strictures on macroscopic evaluation [6–8]. It is impossible to compare different studies owing to the complex and subjective assessment procedure and the lack of a standardized scoring system for histological fibrosis. Hence, establishing a simple and reliable histopathological method to quantify bowel fibrosis in patients with CD is crucial for developing a novel, targeted therapy and interpreting non-invasive imaging results.

We created a new histopathologic index, the collagen area fraction, to quantify the amount of collagen fiber that is deposited in the bowel wall, using a simple imaging manipulation process with the Image J software. The result of our study showed that the collagen area fraction had a good correlation with the histological fibrosis score for the assessment of bowel fibrosis. However, in some samples, there were still incongruous changes that manifested as a high fibrosis score with a low collagen area fraction, whereas in others, there was a high collagen area fraction and a low histological fibrosis score. The histopathological scoring system that was selected as the standard for comparison in our study grades bowel fibrosis according to the deposition depth of collagen fiber. Although the deposition depth of collagen fiber contributes to the formation of CD-associated fibrostenosis, the deposited amount of collagen fiber that is described by the collagen area fraction may play a more important role in the progression of disease, and our index better reflects the severity of bowel fibrosis than the histological fibrosis score. This hypothesis was partly confirmed by our other results, in which the collagen area fraction demonstrated a slightly stronger relationship with the formation of CD strictures than the histological fibrosis score did. The collagen area fraction characterizes the severity of bowel fibrosis from a new perspective. Moreover, it is more suitable as a reference standard for some non-invasive radiological studies (e.g., magnetization transfer in magnetic resonance imaging and ultrasonic elastography) in patients with CD because the parameters that are derived from these novel imaging modalities are mainly affected by the changes in the amount of collagen fibers [9, 15]. Moreover, our result indicated that when the collagen area fraction was ≥0.45, fibrostenosis is highly likely to develop in the pathological bowel segment. The collagen area fraction, which can be only used on surgical specimens, may be a promising biomarker to predict if some CD patients are at a high risk of developing fibrostenosis.

Our study has some limitations. First, we did not analyze the details of the tissue properties in the fibrotic bowel wall, such as smooth muscle hyperplasia or hypertrophy, which were recently reported to play an important role in CD stenosis [5]. However, the aim of our study was to develop a simple and reliable histopathological index that can be a unified reference standard to compare existing studies and quantify bowel fibrosis in CD patients. Second, the semi-quantitatively histopathological scoring systems that were described in prior studies [6–11] have been the best reference standards for bowel fibrosis in CD patients before our study. Hence, the difference between the collagen area fraction and histological fibrosis score for assessing bowel fibrosis could not be compared directly due to the lack of a better third-party reference standard. Third, we did not score the bowel fibrosis at multiple sites in the same section. Hence, the reproducibility of intra-lesions scores using the semi-quantitatively histopathological scoring systems could not be determined.

## Conclusions

In conclusion, the collagen area fraction is a simple and reliable index to reflect the amount of collagen fiber that is deposited in the bowel wall and quantify the severity of bowel fibrosis in patients with CD-associated fibrostenosis. It can be used as an objective reference standard to help compare the results of different CD studies.

## Data Availability

The dataset supporting the conclusions of this article is available from the corresponding author on reasonable request.

## Abbreviations

AUC: The areas under
CD: Crohn’s disease
CDAI: Crohn’s Disease Activity Index
CRP: C-reactive protein
ESR: Erythrocyte sedimentation rate
ICC: Intra-class correlation coefficient
LoA: Limits of agreement
ROC: Curves
ROC: Receiver operating characteristics
